# Physical wedge as a tool for radiochromic film calibration

**DOI:** 10.1016/j.zemedi.2023.05.008

**Published:** 2023-06-29

**Authors:** Stevan Pecić, Miloš Vićić, Ivan Belča, Strahinja Stojadinović, Borko Nidžović, Ljubomir Kurij, Slobodan Dević

**Affiliations:** a Faculty of Physics, University of Belgrade, Studentski trg 12-16, Belgrade 11000, Serbia; b Department of Radiation Oncology, University of Texas Southwestern Medical Center, Dallas 75390, TX, United States; c Institute of Oncology and Radiology of Serbia, Pasterova 14, Belgrade 11000, Serbia; d University Clinical Center of Serbia, Center for Neurooncology, Gamma Knife, Koste Todorovića 4, Belgrade 11000, Serbia; e Medical Physics Unit, McGill University, Montreal H4A 3J1, QC, Canada; f Department of Radiation Oncology, SMBD Jewish General Hospital, Montreal H3T 1E2, QC, Canada

**Keywords:** Radiochromic film dosimetry, EBT3, Calibration, Dose gradients, Physical wedge

## Abstract

Reliable calibration is one of the major challenges in using radiochromic films (RCF) for radiation dosimetry. In this study the feasibility of using dose gradients produced by a physical wedge (PW) for RCF calibration was investigated. The aim was to establish an efficient and reproducible method for calibrating RCF using a PW. Film strips were used to capture the wedge dose profile for five different exposures and the acquired scans were processed to generate corresponding net optical density wedge profiles. The proposed method was compared to the benchmark calibration, following the guidelines for precise calibration using uniform dose fields. The results of the benchmark comparison presented in this paper showed that using a single film strip for measuring wedge dose profile is sufficient for estimating a reliable calibration curve within the recorded dose range. Furthermore, the PW calibration can be extrapolated or extended by using multiple gradients for the optimal coverage of the desired calibration dose range. The method outlined in this paper can be readily replicated using the equipment and expertise commonly found in a radiotherapy center. Once the dose profile and central axis attenuation coefficient of the PW are determined, they can serve as a reference for a variety of calibrations using different types and batches of film. This investigation demonstrated that the calibration curves obtained with the presented PW calibration method are within the bounds of the measurement uncertainty evaluated for the conventional uniform dose field calibration method.

## 1 Introduction

The radiochromic film (RCF) is a suitable dosimeter for 2D dose measurements in high-energy photon beams due to its tissue equivalence, high spatial resolution, and minimal energy dependence [1]. When paired with a flatbed color scanner, it can create a film dosimetry system capable of dose measurements with an uncertainty of 2% or better [2]. Film response to dose is evaluated by changes in light transmission or reflection properties, usually indicated by differences in optical density. The film dosimetry system is influenced by multiple factors affecting dose response, requiring individual calibration for each film batch, or even for a new box from the same batch, to ensure accurate dose determination. This procedure typically involves exposure to homogeneous fields at several different dose levels. Calibration quality is primarily determined by the chosen calibration function and number of points, but also influenced by the active medium’s inhomogeneity, scanning technique, and numerical processing of scans.[3], [4].

Dose gradients offer an alternative to multiple homogeneous radiation fields, providing numerous calibration points in a single film exposure. They are primarily implemented as specifically designed plans [5], [6], but they can also be produced by using a beam modifying filter. A method that utilizes dose gradients by measuring the same dose profiles with different monitor units and estimating the dose points through interpolation has been proposed by Rosca [7]. The method applies stereotactic cone irradiations to a single film, generating the calibration curve and dose profile simultaneously, thereby reducing uncertainties and streamlining calibration and kernel determination for any stereotactic detector. Similar calibration technique has been proposed by Resch et al. [6], where calibration function is optimized by utilizing constant dose profile ratio as an optimization constraint. The proposed dose ratio method could decrease the number of measurements necessary for obtaining an EBT3 film calibration function, while retaining the same level of accuracy as the conventional method. Although both promising alternatives with the potential to accelerate RCF calibration (without compromising accuracy), these methods lack the simplicity of implementation needed for routine usage.

This work presents a gradient-based RCF calibration method using a physical wedge (PW) for dose gradients, similar in aim to previous attempts, but innovatively applying a standard radiation field well established and discussed throughout literature [8], [9], [10], [11]. As demonstrated in this study, if the reference dose profile and attenuation on the central axis (CAX) of the PW are reliably measured, only one film exposure is sufficient for the accurate calibration over the range of recorded doses. The use of a PW is straightforward, requiring no special plans and utilizing units widely available in clinical practice. Once recorded, the normalized dose profile and the attenuation coefficient for a particular PW can be used to calibrate a batch of RCF.

## 2 Materials and methods

### 2.1 Benchmark film calibration

For validation, the proposed PW calibration method was compared to the standard calibration of the GafChromic® EBT3 film batch obtained as the benchmark calibration (BC). To minimize the error produced by the film’s curvature with respect to the scanner bed [12] and the lateral response artifact of the flatbed scanner, a special transparent *PMMA* film positioner was fabricated for this purpose. Calibration was performed by exposing ten 2.54cm×20.32cm film strips to homogeneous 6 MV 15cm×15cm photon fields using Elekta Versa HD linear accelerator. Output of the linear accelerator was verified prior to the film irradiation using a constancy measurement ion chamber cross-calibrated with secondary standard ion chamber (PTW – TN30013), following the IAEA TRS-398 [13] reference dosimetry protocol. The photon fields were designed to deliver doses ranging from 1 to 5.5 Gy, in increments of 0.5 Gy. The irradiation and scanning procedures were carried out according to the protocol by Devic et al. [14]. For the irradiation, film strips were placed in a solid water RW3 Slab Phantom (PTW, Freiburg, Germany) at a 100cm SSD and 5cm depth. Before scanning the film strips, the Epson 12000XL flatbed scanner lamp was warmed up by performing 5 preview scans and 5 empty bed scans. After warming up, each strip was scanned 5 times before and after irradiation, with a resolution of 127 dpi in transmission mode. Zero light (background) scans were obtained by scanning an improvised zero light strip - an aluminum foil sandwiched between two papers, cut to the size of the used film strip. Obtained film scans were saved as RGB TIFF images, median filtered and averaged, from which the resulting average scan was subsequently wiener filtered with a 7×7 kernel. The 1mm×1mm ROI was chosen for calculation of the mean red channel pixel values, from which the net optical density (netOD) was derived following the expression 1:

(1) $$\mathrm{netOD}={\mathrm{OD}}_{\exp}-{\mathrm{OD}}_{\mathrm{unexp}}=\log_{10}\frac{{\mathrm{PV}}_{\mathrm{unexp}}-{\mathrm{PV}}_{\mathrm{bckg}}}{{\mathrm{PV}}_{\exp}-{\mathrm{PV}}_{\mathrm{bckg}}}$$

where ${\mathrm{PV}}_{\exp}$ and ${\mathrm{PV}}_{\mathrm{unexp}}$ represent pixel values of the exposed and unexposed film strips, respectively, while ${\mathrm{PV}}_{\mathrm{bckg}}$ represents pixel value of the zero-light (background) strip. Corresponding netOD uncertainty was calculated following the error propagation expression:

(2) $$\sigma_{\mathrm{netOD}}=\frac{1}{\ln10}\sqrt{\frac{{\left(\sigma_{{\mathrm{PV}}_{\mathrm{unexp}}}(D)\right)}^{2}+{\left(\sigma_{\mathrm{bckg}}(D)\right)}^{2}}{{\left({\mathrm{PV}}_{\mathrm{unexp}}(D)-{\mathrm{PV}}_{\mathrm{bckg}}(D)\right)}^{2}}+\frac{{\left(\sigma_{{\mathrm{PV}}_{\exp}}(D)\right)}^{2}+{\left(\sigma_{\mathrm{bckg}}(D)\right)}^{2}}{{\left({\mathrm{PV}}_{\exp}(D)-{\mathrm{PV}}_{\mathrm{bckg}}(D)\right)}^{2}}}$$

The film calibration curve was established by fitting the measured points to an analytical form:

(3) $$D=a\cdot \mathrm{netOD}+b\cdot {\mathrm{netOD}}^{n}$$

using the “Levenberg–Marquardt” quasi-Newton minimization algorithm, where a and b are free parameters and n=2.5 is fixed for this measurement.

An earlier paper [2] presented an evaluation of the uncertainty in radiochromic film dose measurements, which differentiates between the uncertainty resulting from the calibration curve fit and that from the experimental component. In this section, we briefly summarize the outcomes from this dose uncertainty evaluation, which resulted in the relative experimental uncertainty (4) and the relative fit uncertainty (5):

(4) $$\sigma_{D_{\exp}}(\%)=\frac{\sqrt{{\left(a+n\cdot b\cdot {\mathrm{netOD}}^{n-1}\right)}^{2}\cdot \sigma_{\mathrm{netOD}}^{2}}}{D}\cdot 100\%$$

(5) $$\sigma_{D_{\mathrm{fit}}}(\%)=\frac{\sqrt{{\mathrm{netOD}}^{2}\cdot \sigma_{a}^{2}+{\mathrm{netOD}}^{2n}\cdot \sigma_{b}^{2}}}{D}\cdot 100\%$$

where $\sigma_{a}$ and $\sigma_{b}$ denote the uncertainties of the fitting parameters. Lastly, the total relative uncertainty for the BC dose measured using the aforementioned formalism for the functional form specified by Eq. (3) is determined as:

(6) $$\sigma_{D}(\%)=\sqrt{\sigma_{D_{\exp}}^{2}+\sigma_{D_{\mathrm{fit}}}^{2}}$$

Deviations of the calibration curves obtained by the presented PW methods from the BC are calculated for each of the BC measurement points as:

(7) $$\frac{\Delta D}{D}(\%)=|\frac{D_{\mathrm{BC}}-D_{\mathrm{PW}}}{D_{\mathrm{BC}}}|\cdot 100\%$$

where $D_{\mathrm{BC}}$ denotes a dose measurement obtained by BC and $D_{\mathrm{PW}}$ denotes a dose measurement obtained by PW calibration.

### 2.2 Wedge reference measurement

The reference wedge dose profile used in this work was produced by motorized 60°PW. The measurements were done using a 6 MV photon energy with open 15cm×15cm fields. Reference inline and crossline profiles were measured in a standard 3D water tank using CC13 ionization chambers pair (IBA Dosimetry, Schwarzenbruck, Germany). Also, the attenuation on the CAX for the particular PW was determined by measuring the absolute dose at the isocenter with a PW in place, following the previously mentioned protocol for determination of the absorbed dose [13]. The measured PW attenuation coefficient on the CAX at a 100cmSSD and 5cm depth was 0.2654. Attenuation was measured for $D_{\mathrm{CAX}}=2\;\text{Gy}$ and found to be consistent within the used dose range. The attenuation coefficient was used to recalculate monitor units corresponding to CAX doses of the designed wedge fields.

### 2.3 Wedge optical density profiles

Optical density profiles for PW used were obtained by exposing 5 film strips (identical to the BC film strips) to fields corresponding to absorbed doses of 1,2,3,4 and 5 Gy on the CAX at depth of 5 cm. The films were positioned using the lasers in the therapy room in the identical solid water phantom setup used to irradiate the calibration strips at a depth of 5 cm in the SSD setup. It is of crucial importance to position films inside the phantom without rotating them with respect to the beam (as well as the film placement on the scanner bed with film edges parallel to scanner edges), having in mind that film rotations could lead to recording altered wedge profiles. Films were irradiated with the same field as the wedge reference profile and scanned identically as the previously described BC film strips. The resulting scans were processed in the same way as the calibration strips, from which the corresponding netOD maps were calculated using the Eq. (1). Scans representing BC and wedge fields were processed using an in–house script written in Matlab for this purpose.

As a denoising step, 25 central lines on the wedge axis were extracted from the netOD map and averaged. The edges of the field were segmented from the averaged pixel array using a numerical gradient [15]. A sanity check of the field extraction was performed by calculating the length of the extracted netOD profile in units of absolute length. If the film is placed below the isocenter, the profile should be slightly longer than the given collimator aperture for that axis, taking into account the effect of beam divergence with depth. For clarity, a monotonically increasing/decreasing series of measurement points were used, introducing another segmentation that extracted the region from 20% to 80% of the profile length, effectively removing the remains of the penumbra. Finally, the resulting optical density profile was smoothed by the moving mean algorithm.

**Figure 1:**
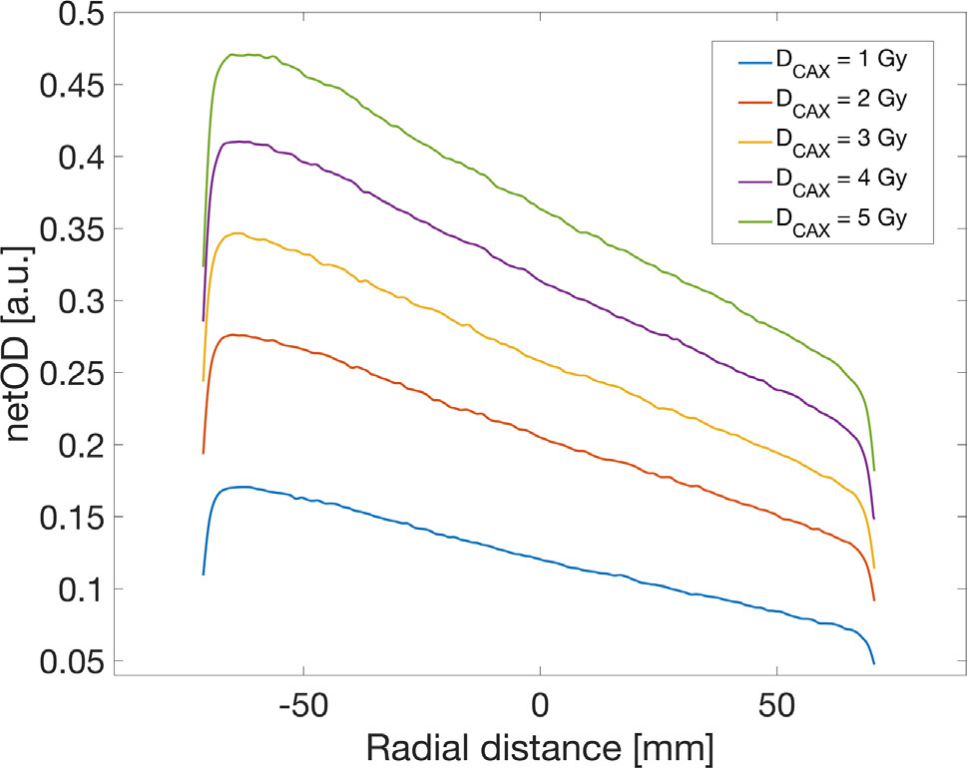
Film-captured wedge netOD profiles.

Wedge fields obtained by film measurement are presented in Fig. 1. These measurements were used as an input to the fitting procedure, with the monotonically decreasing part extracted from the profile.

**Figure 2:**
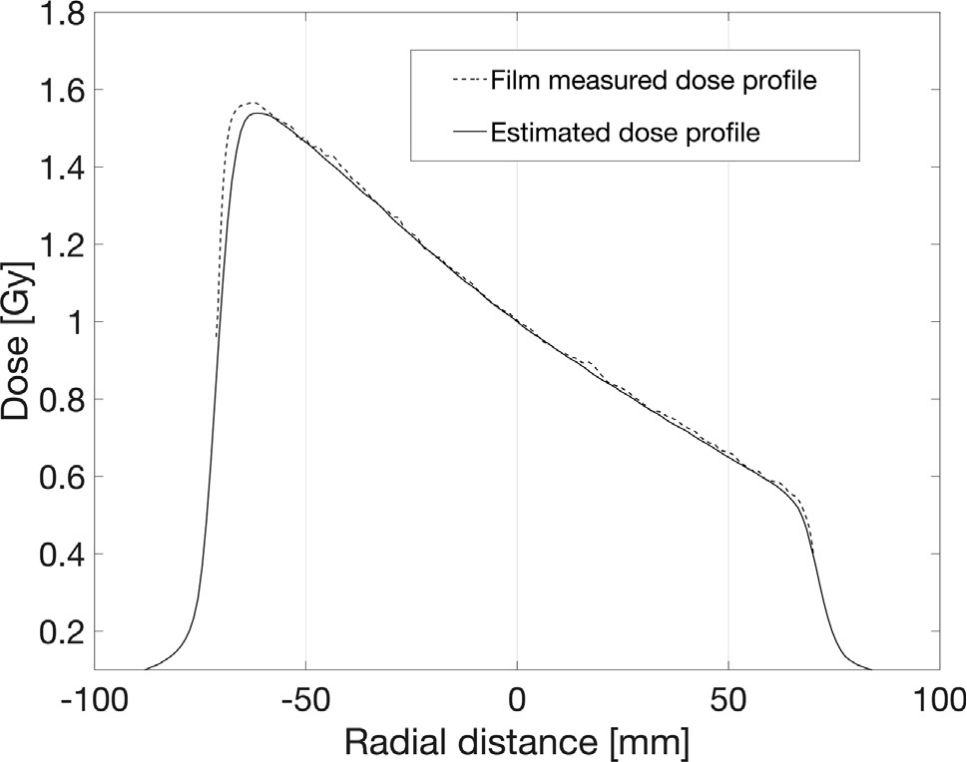
Wedge dose profile estimation for $D_{\mathrm{CAX}}=1\;\text{Gy}$.

Corresponding dose profiles are estimated as the CAX dose multiple of the reference dose profile. In other words, the dose profiles are self-similar and represent a multiple of the reference wedge profile, normalized to $D_{\mathrm{CAX}}=1\;\text{Gy}$. As a proof of concept, the dose profile estimated by multiplication of the normalized wedge profile was compared to a measured profile calculated from the measured netOD wedge profile and BC. The estimated and measured profiles for $D_{\mathrm{CAX}}=1\;\text{Gy}$ are shown in Fig. 2. The presented data show agreement within 2% with the BC which serves as a validation for the dose profile estimation. The small deviation from the BC is a consequence of the CAX attenuation uncertainty as well as dose measurement uncertainty.

## 3 Results

### 3.1 Dose calibration fit

**Figure 3:**
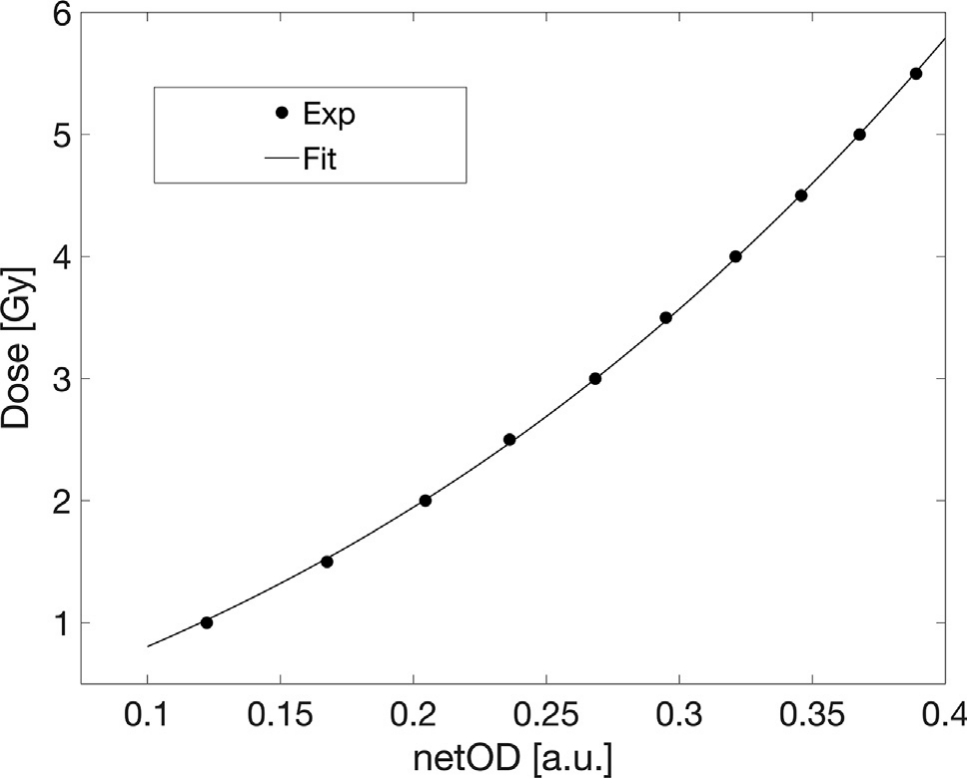
The Benchmark Calibration (BC) fit.

**Figure 4:**
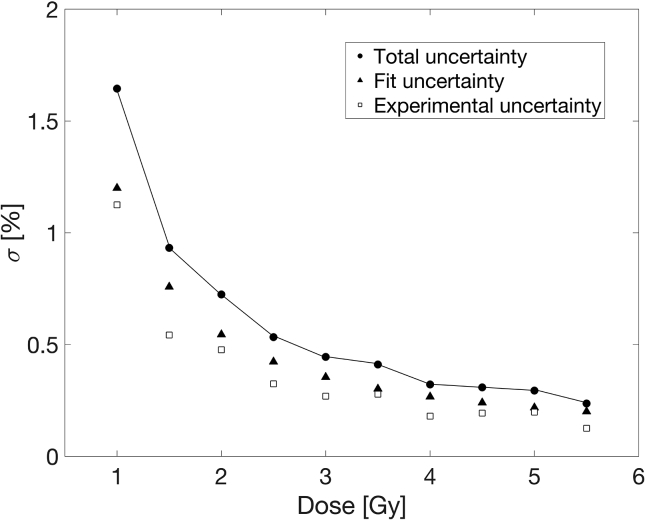
The Benchmark Calibration (BC) uncertainty distribution. Component experimental and fit uncertainties were calculated following the expressions (4) and (5). Total uncertainty is the quadrature-sum of the component uncertainties and is explicitly given by the expression (6).

Following the described procedure for benchmark calibration, the obtained BC curve is depicted in Fig. 3. Dose uncertainty was calculated following the uncertainty analysis described in Devic et al. [14] and the corresponding distributions of component experimental and fit uncertainties, as well as the total measurement uncertainty are shown in Fig. 4. As shown in this Figure, uncertainties of the BC were less than 2% for doses above 1 Gy.

**Figure 5:**
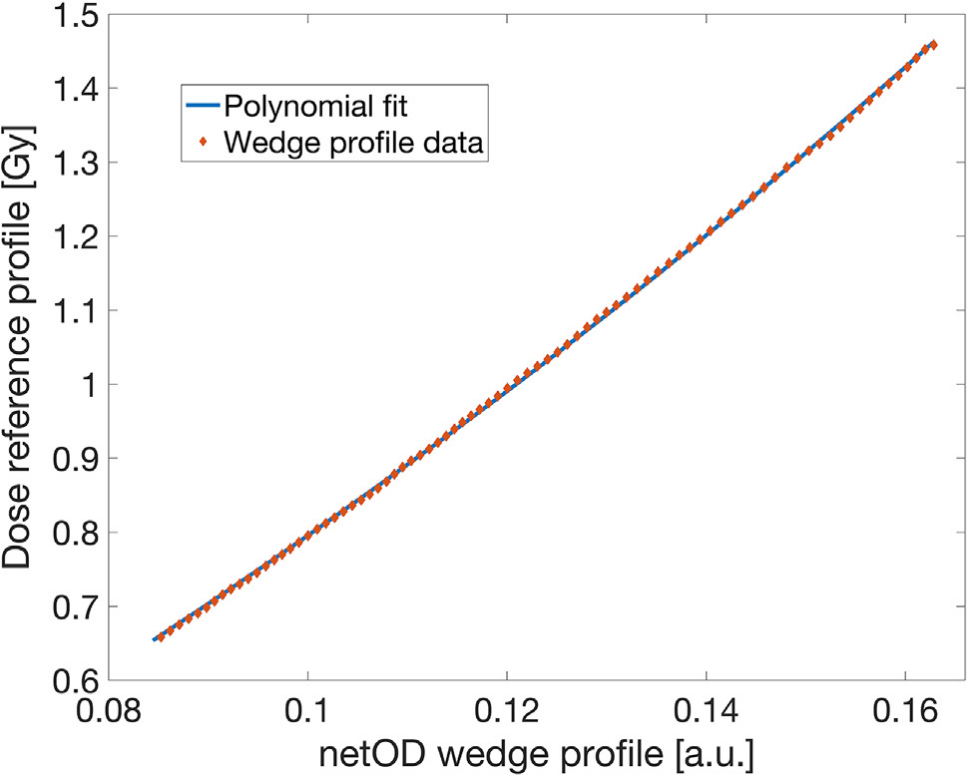
Dose profile as a function of the measured netOD profile. The polynomial analytical form fit of measured points shows dose calibration function of the used film batch.

Preprocessing of dose and netOD profiles resulted in more than 500 measurement points for a single dose gradient. Measured dose reference profile as a function of the film-captured netOD profile for the $D_{\mathrm{CAX}}=1\;\text{Gy}$ is presented in Fig. 5. The points in the presented graph directly portray the dose-netOD relationship, therefore by using a standard nonlinear curve fitting algorithm, a calibration curve was obtained.

**Figure 6:**
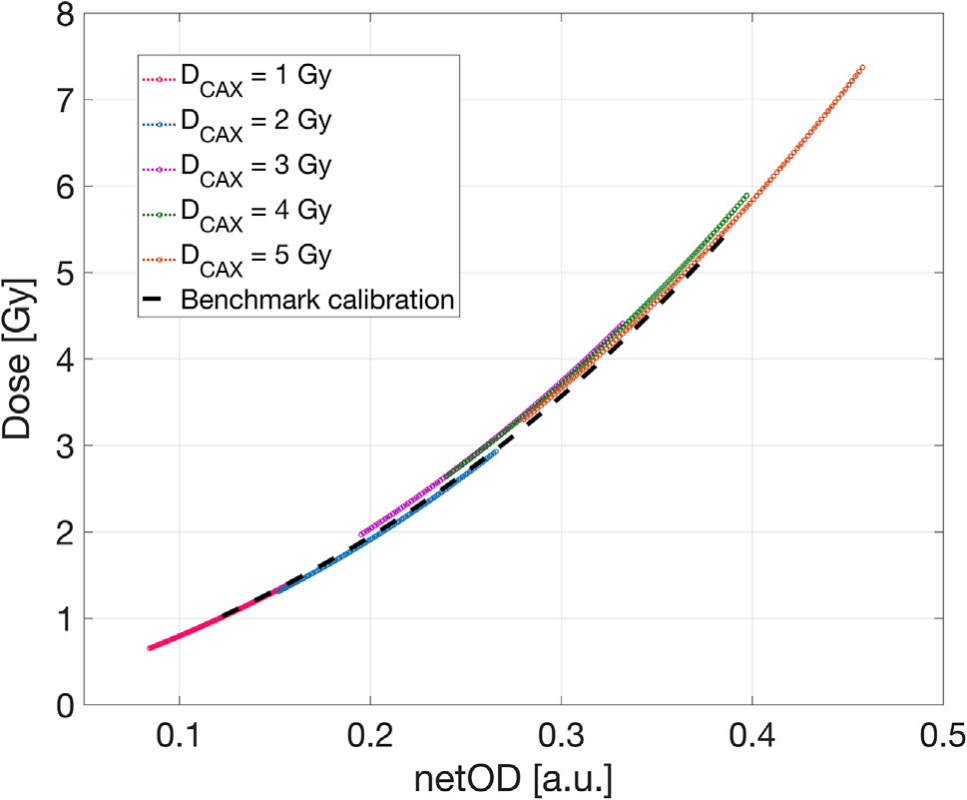
Calibration curves for $D_{\mathrm{CAX}}=[1,2,3,4,5]\;\text{Gy}$ wedge fields in comparison with BC fit.

**Figure 7:**
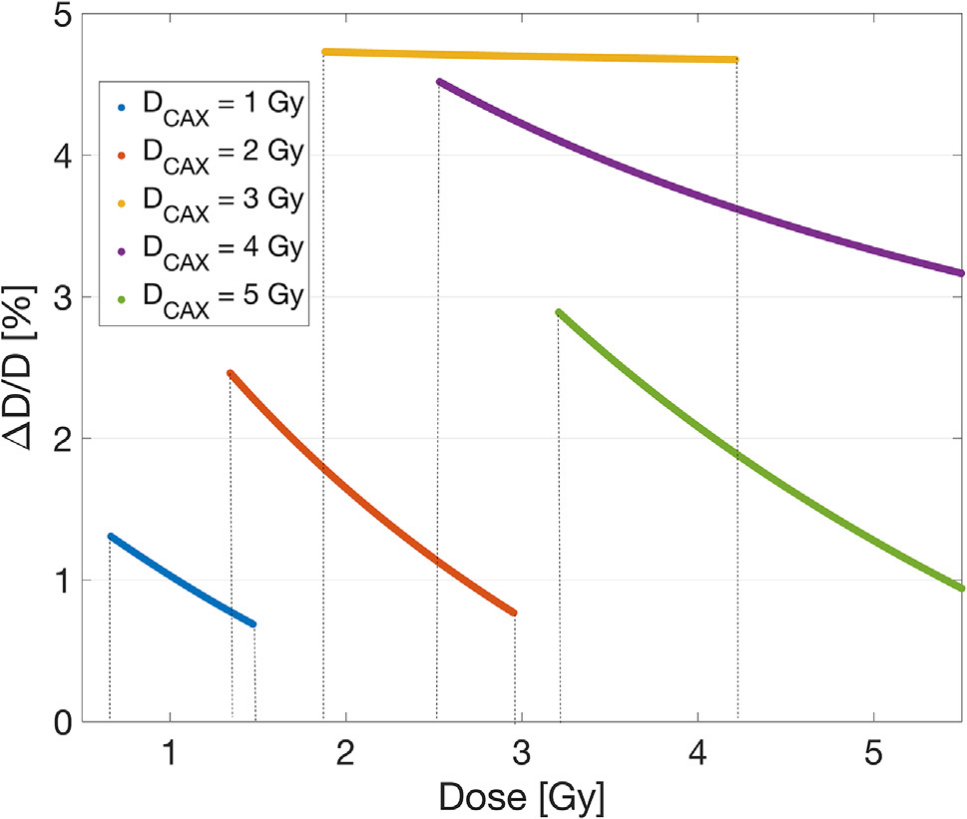
Deviation of the wedge fit from the BC fit.

**Figure 8:**
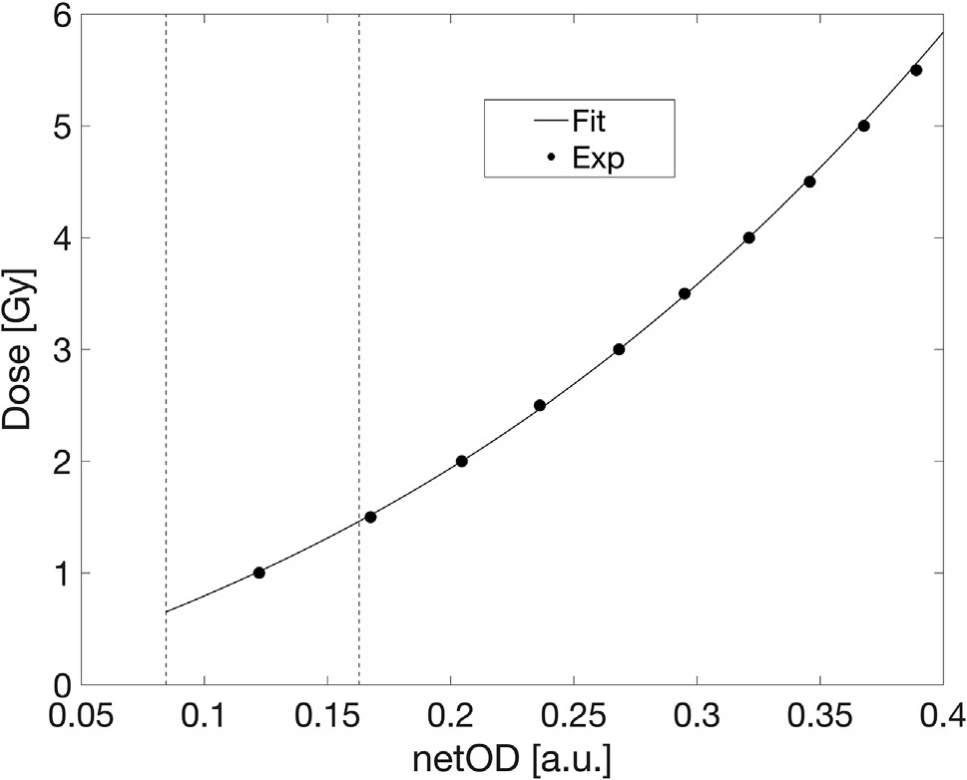
Extrapolated fit for the wedge $D_{\mathrm{CAX}}=1\;\text{Gy}$ gradient. Dashed lines represent boundaries of the acquired wedge dose gradient.

Wedge fit comparison with BC curve is presented in Fig. 6. Deviation of the obtained fits for $D_{\mathrm{CAX}}=1,2,3,4$ and 5Gy from the BC points is presented in Fig. 7. The results demonstrate that the deviation was below 3% for the gradients with $D_{\mathrm{CAX}}=1\;\text{Gy},D_{\mathrm{CAX}}=2\;\text{Gy}$ and $D_{\mathrm{CAX}}=5\;\text{Gy}$ and below 5% for $D_{\mathrm{CAX}}=3\;\text{Gy}$ and $D_{\mathrm{CAX}}=4\;\text{Gy}$. This analysis shows the deviation of the wedge estimated curve relative to the BC curve was within the 2% estimated dose uncertainty presented in Fig. 4, which proves that the wedge field calibration can provide a fit within the limits of benchmark estimated uncertainty (see Fig. 8).

### 3.2 Fit extrapolation

The dynamic range of the wedge dose gradients is usually insufficient for calibration at the dose range relevant to typical clinical utilization. To extend the range of calibration doses with a single exposure, extrapolation of the calibration curve was tested. The extrapolation was achieved by fitting a narrow range netOD gradient range and subsequently constructing a calibration curve over the extended BC domain. Extrapolated fits deviate from the BC by less than 2% for the $D_{\mathrm{CAX}}=1\;\text{Gy}$. We found that the polynomial analytical form was the most reliable for fit extrapolation, in contrast to the rational analytical forms suggested in the literature which in our case failed to provide acceptable fit quality in the extrapolated region.

### 3.3 Using multiple gradients

The scarce dynamic range of the wedge-produced dose gradient can be also compensated by multiple film exposures providing multiple dose gradients. As mentioned in the introduction, multiple dose gradients can be utilized for an intercept-based or optimization-based film calibration approach. In this work, the use of multiple dose gradients aims to evenly fill the desired dose range with measurement points, for which purpose concatenation of three profiles into a single profile was performed. As is the case with single gradient calibration, this procedure restricts the method for obtaining calibration curve to simple nonlinear curve fitting.

**Figure 9:**
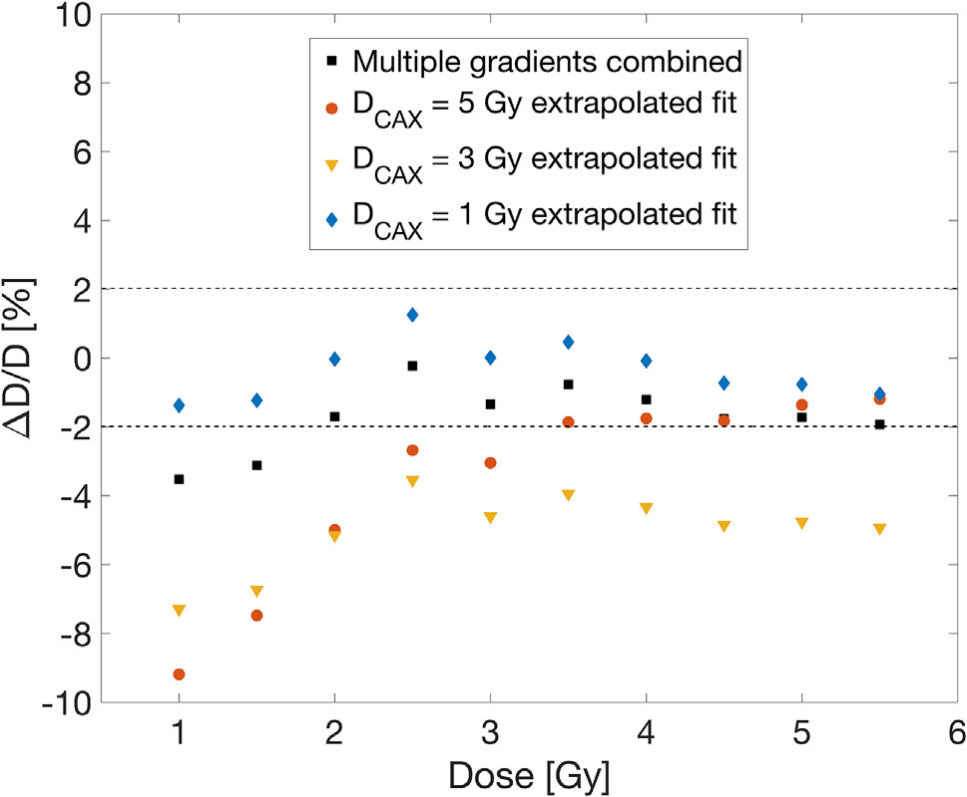
Distribution of uncertainties for the multiple wedge fields fit and extrapolated fit compared to the BC points.

Fig. 9 shows the comparison of dose deviations relative to the BC when using multiple gradients and extrapolated film calibration for $D_{\mathrm{CAX}}=1,3$ and 5Gy. The results demonstrate that the deviation was below 10% for the $D_{\mathrm{CAX}}=5\;\text{Gy}$ and $D_{\mathrm{CAX}}=3\;\text{Gy}$ gradients and below 2% for the $D_{\mathrm{CAX}}=1\;\text{Gy}$ gradient. A single fit with five concatenated dose gradients produced an acceptable dose deviation within 3% from the BC. Compared to a single gradient extrapolated fit, multiple gradients minimized the possibility of the fit being considerably imprecise. However, a good agreement of the $D_{\mathrm{CAX}}=1\;\text{Gy}$ extrapolated fit suggests using a single gradient, but with multiple exposures of the same field, effectively minimizing the error.

## 4 Discussion

The feasibility of PW dose gradients for film calibration was investigated. Using a single film strip for measuring wedge dose profile was proven sufficient for a reliable calibration curve estimation in the desired dose range. Benchmark comparison presented in this paper should not be misunderstood as a measure of uncertainty but rather as a sanity check for the method’s usefulness.

The use of a PW provides a dose gradient that can be measured with high precision. In the future, as a possible acceleration of the method, the dose profile of the PW can be calculated analytically [16], but also evaluated by Monte Carlo simulation. This approach would open the possibility of film calibration using previously measured dose kernel, eliminating the need for independent measurement of the dose profile. The apparent experimental challenge of this method is the positioning of the film inside the phantom. Rotation of the film relative to the center could cause the central field profile to reflect an altered PW dose profile leading to measurement error. Translational sensitivity, on the other hand, is compensated by the gradient-based field segmentation, which extracts the wedge field regardless of the film positioning.

The presented work insists on the accuracy of the single dose gradient captured. The works of Rosca [7] and Resch [6] utilize dose gradient ratios, thereby the conclusions about the necessary number of dose levels for reliable calibration cannot be applied directly to the presented PW method. However, it should be noted that in the case of only one measurement the sensitivity of the measurement increases, which should be compensated by averaging several films exposed with the same dose gradient. This would mostly minimize the fluctuation of the optical density profile caused by the nonuniformity of the film’s sensitive layer. Nonetheless, as it has been shown that the sensitive layer thickness uniformity for newer film models (EBT3 or newer) is improved to the point of optical density being justifiably replaced by raw pixel values [17], the authors believe that this correction would give diminishing returns.

The analytical form of the fit function can make a strong influence on the extrapolation fit quality. At the time of writing this paper, the response of RCF is still a subject of general discussion and the papers [18], [19], [20], [21] propose several interesting approaches to dose response modeling. An analytical model which faithfully describes the underlying mechanism of radiation-induced polymerization, such as the work presented by Rodríguez et al. [21], is expected to give the best results, but it is not found in widespread use so far.

For the optimal coverage of the deemed dose range, PW calibration can be further optimized when using multiple gradients. The quantity that determines the dynamic range of a given PW is the angle of the wedge. It can be defined as the angle through which an isodose is tilted at the CAX of the beam, at a specified depth [22]. The results presented in this paper were obtained using a PW with an angle of 60°at the depth of 5cm, which is the maximum angle of PWs found in clinical use. Using a PW with smaller angle would result in narrower dose coverage generated in a single exposition, but this disadvantage can be overcome by using multiple gradients or fit extrapolations. Also, for a given wedge, the dose ratios can be optimized to evenly cover the desired calibration dose range. Given the exponentially decaying nature of the dose uncertainty when using film, calibration quality can be improved with a larger number of points in the region of lower optical densities. Finally, the scanning resolution could be optimized in the direction of the best fit, considering that increasing the resolution yields a larger number of points. Higher resolution, on the other hand, introduces more noise into the optical density profiles which results in a tradeoff between dose range coverage and signal quality. A small amount (such as a thin strip) of film used to acquire wedge field gives the possibility to calibrate each sheet, enabling efficient calibration with minimal loss of material.

Although the practice of radiotherapy has evolved toward the use of beam-modulating wedge simulators [23], the PW as a beam modulator with no moving parts offers reproducibility of the dose profile and attenuation on the CAX, making it the best choice for reproducible measurements. Furthermore, wedge-type applicators are widely available in clinical practice, and medical physicists are familiar with their use. In the general case, it is possible to use any applicator with an irregular lateral or longitudinal profile instead of a PW. This allows for the possibility of using a specially designed wedge-like applicator whose profile and CAX attenuation would be measured in reference conditions and then routinely used for film calibration. With the presented results, it is possible to create a protocol for a simple and precise film calibration.

## 5 Conclusions

Dose gradients produced by PW were investigated and proven useful for RCF calibration. The main advantages of the method are the reliability and reproducibility of the dose profile measured under reference conditions. The described method does not rely on specific mathematical formalism or unusual experimental conditions and can be reproduced using the resources and staff experience of a typical radiotherapy facility. Once recorded, the dose profile and CAX attenuation coefficient of the PW can be adopted as a reference for an unlimited number of calibrations of different batches and film types. Further research on this topic should optimize the presented method by finding optimal measurement parameters. Moreover, the reference dose profile Monte Carlo modeling or analytical wedge field calculation are promising alternatives to reference dosimetry measurements that would accelerate and possibly improve the described method. The final step in the development of the title method would be the formation of a RCF calibration protocol. In the case of the EBT3 film, the presented results indicate that dose measurements obtained using PW calibration may be comparable in quality to those achieved with conventional uniform dose field calibration.

## Declaration of Competing Interest

The authors declare that they have no known competing financial interests or personal relationships that could have appeared to influence the work reported in this paper.
